# Aging and Mechanoadaptive Responsiveness of Bone

**DOI:** 10.1007/s11914-019-00553-7

**Published:** 2019-11-23

**Authors:** Behzad Javaheri, Andrew A. Pitsillides

**Affiliations:** Skeletal Biology Group, Comparative Biomedical Sciences, The Royal Veterinary College, Royal College Street, London, NW1 0TU UK

**Keywords:** Osteoporosis, Loading, Aging, Mechanoadaptation

## Abstract

**Purpose of Review:**

Osteoporosis is an age-related disorder characterized by bone loss and increased fracture susceptibility. Whether this is due to reduced loading in less active elderly individuals or inherent modifications in bone cells is uncertain. We suppose that osteoporosis is nonetheless prima facie evidence for impaired mechanoadaptation; either capacity to accrue new bone declines, or the stimulus for such accrual is absent/can no longer be triggered in the aged. Herein, we provide only sufficient background to enable a focus on recent advances which seek to address such dilemmas.

**Recent Findings:**

Recent advances from innovative high-impact loading regimes emphasize the priming of mechanoadaptation in the aged, such that low-to-moderate intensity loading becomes beneficial. These new findings lead us to speculate that aged bone mechanoadaptation is not driven solely by strain magnitude but is instead sensitive to high strain gradients.

**Summary:**

Impaired mechanoadaptation is a feature of the aged skeleton. Recent advances indicate that novel interventional loading regimes can restore mechanoadaptive capacity, enabling new approaches for retaining bone health in the aged. Innovative exercise paradigms appear to be capable of “hacking” into the osteogenic signal produced by exercise such that low-to-moderate intensity activities may also become more beneficial. Deciphering the underpinning mechanism(s) will also enable new pharmacological intervention for retaining bone health in the aged.

## Introduction

Osteoporosis is most frequently an age-related skeletal disorder. It is characterized by the failure to retain bone mass and by deterioration in bone microarchitecture, which together reduces bone strength and increases susceptibility to fracture. In the USA, over 1.5 million osteoporotic fractures occur annually [1]. The majority of fractures occur in the latter decades of life when rates of bone loss and microarchitectural deterioration are at their greatest [2]. This intimate link between bone aging processes and the pathogenesis of osteoporosis has led to increased basic, clinical, observational, and translational research in recent years on the mechanisms underpinning both age-related bone loss and fragility fractures [3, 4]. Population aging is expected to escalate the prevalence of osteoporosis over the next decades. A better grasp of these mechanisms is crucial if new effective treatments to combat or indeed reverse this age-related decline are to be pinpointed [5].

This goal is centered upon the dynamic, “regenerative” quality of bones that secures its many roles. Bones are not only essential for locomotion, support, and protection of internal structures but is crucial as a reservoir for phosphorus and calcium, important for glucose metabolism, houses the hematopoietic system, and is essential for the function of renal and reproductive systems. To fulfill these mechanical and homeostatic functions, bone must undergo a continual self-regeneration process called remodeling which removes old bones and replaces it with new. This regenerative process plays out on bone surfaces within basic multicellular units (BMUs) [6]. Within each BMU, bone formation by osteoblasts and resorption by osteoclasts are coupled tightly in a delicate balance to maintain mass and strength in the healthy skeleton. This balance in remodeling is known to shift toward less bone formation and greater resorption with aging to culminate in reduced bone strength, osteoporosis, and fractures; can this potential to shift the BMU balance be exploited to find effective and targeted treatments?

Aging is also linked to compositional, architectural, material, and metabolic alterations in both trabecular and cortical compartments. Cancellous bone aging is associated with a reduction in trabecular number, increased spacing, and unaffected or decreased thickness [89, 90]. Aging is also linked to both endocortical resorption and periosteal bone formation, leading to cortical thinning and marrow cavity expansion. In humans, bone mineral density (BMD) thus peaks between 10 and 19 years, with the continued increase in bone mineral content until 30–35 years of age [7]. Although often considered a period during which there is neither net gain nor loss of bone mass, aging processes likely begin soon after the cessation of growth. BMD decreases at the spine and proximal femur in women even before menopause, and bones are indeed lost during early adult years in both men and in women due to the emergence of a negative BMU balance as early as the third decade. This negative balance is due to the combination of intrinsic changes with extrinsic factors.

Intrinsic changes include aging-related modifications in hormone status, gene expression, cell components, and biochemical and vasculature changes. Aging leads to reduced levels of circulating hormones [8–12], basal cell function [13–18], proliferation, and differentiation of stem cells into osteoblasts, as well as diminished osteoblast function and increased apoptosis [19–23]). Aging is also linked to the greater number and activity of osteoclasts in both humans [24] and mice [25, 26]. Bone lining cells and osteocyte density (and lacunar density) diminish with age [27–29], as does osteocyte canaliculi number [30]. Of note, aging may contribute by compromising bones’ regenerative potential, evidenced in the age-related decline in osteogenic progenitor cell numbers in animal models and human samples, and within the bone marrow of adult versus younger animals. Indeed, declining osteogenic cell number coupled with impaired blood vessel formation is considered responsible for the failure in bone regeneration observed in elderly individuals [31]. This may be in keeping with our recent report showing that vascular density is indeed reduced in regions of the aging tibial cortices [32]. It is evident that a multitude of intrinsic factors contributes to age-related osteoporosis and that any combination may represent a target for reversing the effects of aging on mechanoadaptation.

Extrinsic factors include nutrition, comorbid medical conditions, drugs, and of vital significance here an impaired adaptive bone response to loading [33]. Whether age-related bone loss is an adaptation to the reduced loading experienced in less active, elderly individuals or is instead the product of a reduced basal osteoblast or increased osteoclast lifespan, or abnormal osteocyte mechanical signaling is uncertain. Given that the culminating effect is bone loss and increased fracture risk [2], we and others have supposed that age-related osteoporosis is prima facie evidence for impaired response to loading at some level. Thus, either the skeleton’s ability to accrue new bone declines inevitably with aging or the appropriate stimulus for such accrual is absent or can no longer be triggered in the aged skeleton. Herein, we focus on recent (< 3 years) advances that address such dilemmas in age-related bone mechanoadaptive responses.

## Bone Mechanoadaptation

Bones adapt their architecture during life to ensure they are robust enough to withstand the habitual levels of loading to which they are subjected, without accumulation of excessive microdamage or fracture [34]. This functional adaptation, achieved by the processes of (re)modeling induced by loading, leads to modifications in mass as well as architecture to best tailor bone structure to its prevailing mechanical environment [35]. Many clinical studies have confirmed this connection between the mechanical environment and bone structure, mainly in high impact sports [36, 37] and bed rest [38]. These are echoed in animal experiments which exploit externally applied loads [39–44] or unloading [45–47], and in vitro studies where bone cells are challenged by mechanical stimuli or by fluid flow [48–50]. Thus, a lack of weight-bearing due either to prolonged spaceflight or bed-rest leads to decreased bone mass [51–55], while mechanical loading of the skeleton through various types of exercise results in increased bone mass [56–59]. In humans, it has been observed that high impact exercise, such as weight-lifting and gymnastic, generates greater BMD particularly at weight-bearing sites (versus non-athletic age-matched controls). Moreover, professional athletes such as tennis players exhibit a greater BMD in their dominant compared with their contralateral forearm [60, 61]. These human studies add support to the vast range of data from animal experiments which have shown that mechanical loading promotes bone accrual [62, 42, 63–65] while unloading promotes bone loss in vivo [66–68, 47]. Together, these studies establish that the bone’s mechanoadaptive response operates at most life-course stages to match bone architecture to its load-bearing function. The extent to which this occurs in the elderly phase of life is debatable.

## Failure of Mechanoadaptation with Aging

The promotion of exercise-related increases in bone mass in adults [69, 70] is thus somewhat at odds with previous clinical trials in which bone accrual was hardly, if at all, apparent in the elderly [71, 72]. This age-related diminution in mechanoadaptive capacity has been confirmed in animal studies [73–75]. These observations are however open to alternative interpretations: Are they due to inherent age-related changes in bone formation and resorption activities or drifting bone cell behavior, an impairment in the ability of bone cells to transduce mechanical stimuli into biochemical signals or due to modified local microenvironmental cues? Might this failure be underpinned by micro-structural changes such as modified osteocyte lacunar organization or directional orientation, or changes in the number/length of inter-canniculular connecting dendrites [76, 77]? Irrespective of its basis, many have proposed that this failure in functional adaptation is responsible for the osteoporosis which develops in the aged skeleton [78, 16, 79–81, 34, 82, 83, 63, 84].

The mechanisms underpinning this age-related decline in mechanoadaptation have been the subject of numerous recent studies. Galea et al. (2017) explored the transcriptomic changes linked with this age-related impairment to find a down-regulation of genes with enrichment in MAPK, Wnt, and cell cycle pathways and closest association with down-regulation of the E2F1 transcription factor, which also exhibited diminished levels of protein expression in osteocytes with aging. On the other hand, aged bones exhibited up-regulation of genes enriched for carbohydrate metabolism and for TNFα and TGFβ superfamily components. Evaluation of transcriptomic changes induced by applied mechanical loading revealed rapid and sustained, yet distinct transcriptional responses in young and old bones, characterized by up-regulation of genes predominantly related to proliferation in the young and their down-regulation by loading in the aged; conversely, genes linked closely with bioenergetic pathways were down- and up-regulated in the young and aged, respectively. The authors concluded that loading engenders a more sustained gene response in the young [85]. In line with the aforementioned age-related down-regulation of Wnt pathway genes, previous studies had described load-induced Wnt pathway activation as well as a reduction in its negative inhibitor, Sclerostin (product of Sost gene), in response to in vivo loading in younger mice [86••, 44]. Together these data suggest that aging is accompanied by less robust or aberrant activation patterns for load-related pathways with known mechanoadaptive function.

A recent study by Holguin et al. (2016) reported that tissue-level indices of bone formation, observed after only 1 or 5 days of loading, are also less pronounced in the aged. Likewise, 3–5 days of loading produced a smaller up-regulation of bone formation-related genes (e.g., Col1a1) in the old than in young-adult mice. This was supported by experiments showing a failure to elicit the prototypical Sost down-regulation in response to repetitive bouts of loading in aged mice, which accompany the load-related osteogenic response observed in young counterparts. This study additionally found that early and sustained load-induced up-regulation of Wnt1/7b in the bones of young-adult mice was much less robust upon aging. The authors concluded that the reduced bone anabolic response to loading in aged mice includes a failure to sustain Wnt activity [87••], attributing this to a lower sensitivity to repeat loading in the aged.

This notion of an age-related reduction in load sensitivity appears to be confirmed by two recent studies investigating combined in vivo mechanical loading and pharmacotherapy. Meakin et al. (2017) reported accrual of the cortical bone in response to intermittent PTH treatment (iPTH), which additionally generated positive load-related osteogenic interactions in young mice. Aged mice, in contrast, exhibited no positive load-iPTH interaction in the cortical compartment [88] suggesting that aged bones have reduced sensitivity to pharmacotherapy and mechanical loading. With view to better ascertain the role of osteoclasts in aged bones, Naruse et al. (2016) compared the efficacy of alendronate as a means of restricting bone loss in aged female rats, which were sedentary, estrogen-deficient, or both. Rats were either restrained in a sitting position or allowed free cage activity, with or without alendronate administration for 8 weeks after a 5-week-long preceding period after ovariectomy or sham surgery. They found that alendronate failed to protect against a lowering of bone breaking stress engendered by “sitting”, irrespective of prior ovariectomy, and that “sitting” also increased the mineral-to-matrix and carbonate-to-phosphate ratios, also seen in aged bones, which results in fragility-related deficits in both the quality and geometry of the cortical bone. They concluded that bisphosphonates may provide therapy best suited to osteoporotic aged individuals whose daily activity is not limited [89]. These data also imply that some protection afforded by the anti-osteoclastic activity of bisphosphonates in aging may require simultaneous interaction with a load-derived stimulus.

These studies align with earlier findings indicating that cells in aged bones acquire a reduced mechanosensitivity and an attenuation of the strain signal derived from loading due to increased stiffness, which may contribute to the pathogenesis of age-related bone loss [78, 90–96]. These studies also found that mechanoadaptive responses, most likely coordinated by osteocytes, depend on load magnitude, frequency, and rate that are more effective in young than in aged bones. These studies also report that periosteal surfaces are less load-responsive than those observed endocortically and that the latter decline with aging. However, many questions remain: Does the bone accumulate age-related history of loading? Are mechanoadaptive responses coordinated solely by locally load-induced maximum strains or by more complex integration of strain patterns? Are mechanoadaptive responses interrupted when the load stimulus is brought within a certain threshold? How is bone formation coordinated to focus it directly to regions of high strain stimulus or elsewhere? Answers will provide new paradigms for controlling bone mass via interventional trials for the treatment of age-related bone loss.

## Intervention Studies

Several interventional studies in animals and humans aimed at interrogating the basis of this age-related failure of mechanoadaptation and its potential priming. De Souza et al. (2017) hypothesized that habitual load exposure underpins the aged bone’s lower mechanoadaptive capacity. To test this, they performed sciatic neurectomy in both aged mice and adult mice that were allowed to age in order to induce disuse (SN-disuse) prior to load application. They reported that (i) in agreement with previous studies, load responses become defective in aged mice and are restored by the potential priming effect of short-term SN-related disuse; (ii) prolongation of functional SN-disuse further augments load responses in cortical bone but, (iii) blunts any rescue in the trabecular compartment elicited by short-term disuse. These findings indicate that lengthening the disuse period more effectively primes age-related mechanoadaptive responses in the cortical bone but abolishes the beneficial effects of short-term SN-related disuse in the trabecular compartment. In a follow-up study, Piet et al. (2019) reported that the synergistic effect of SN and loading in aged mice is likely due to increased number of osteoclasts and osteoblasts on the endosteal surfaces [97]. These data point to the requirement for a combined approach for restoring mechanoadaptive response in the bones of aged mice [47]. In a more recent study, Cunningham et al. (2018) examined whether aged rats would lose little if any bone with disuse compared with adult rats and if so, whether aged rats would exhibit diminished recovery following treadmill exercise. Consistent with this notion, adult but not aged rats showed a marked decrease in trabecular bone volume following unloading—imposed through the hindlimb elevation. They found however that aged bones were also less responsive to reloading [98]. The different conclusions drawn in the studies of de Souza et al. (2017) and Cunningham et al. (2018) may be due to the distinct approaches to both the induction of disuse (neurectomy vs hindlimb suspension) and the method used to reload the skeletal elements (localized tibial loading vs treadmill exercise).

Exercise is one of the primary modifiable factors linked to bone health, such as improved bone mass and geometry [99]. However, the experimental evidence supporting influence of exercise on bone structure and strength in older people is scarce and somewhat conflicting. A previous meta-analysis found no significant exercise effects on bone strength [100]. Since then, studies on middle-aged and older people have found positive, site-specific effects on the proximal femoral bone mass after impact training [101••] but no effects on the mid-femoral or mid-tibial structure and strength after strength training or combined strength and impact training [102, 103]. In this light, recent exercise interventions have implemented varied types of exercise to restore (prime) the age-related decline in bone mass. For example, Gombos et al. (2016) examined serum bone turnover markers associated with anabolic effects of exercise in response to a single session of resistance exercise in aged participants with low bone mass. Measurement of bone-specific alkaline phosphatase, carboxy-terminal cross-linked type I collagen telopeptide (CTX), and serum Sclerostin levels before and immediately after a single exercise intervention revealed significant decreases only in serum CTX levels, suggesting that such regimes can exert direct bone mechanoadaptive responses in aged individuals [104].

Bolam et al. (2016) also sought to identify optimal exercise strategies to counteract age-related bone loss. They examined the scale of the osteogenic effect on BMD and evaluated safety and feasibility of a program of upper body resistance exercise combined with either of two regimes of impact-loading (high/moderate, 80/40 jumps/session) in middle-aged and older men. The 9-month intervention involved 4 sessions/week: 2 supervised clinic-based and 2 home-based. This study reported significant shifts in total hip and trochanter BMD, with a decline in control and moderate jumping groups but preservation in hip BMD in the high loading group. These data indicate that while impact-loading exercise can be safely undertaken in middle-aged and older men to partly protect against age-related bone loss, current recommended regimes fail to elicit significant BMD improvements [105]. Another study by Seidelin et al. (2017) showed however that 12 weeks of twice-weekly floorball training induced superior performance, leaner body mass, and greater total leg BMD in both pre/recently postmenopausal women [106]. Together, these new data indicate that novel exercise regimes are being developed that exploit our knowledge of bones’ load-related mechanoadaptive responses in the aged.

In a randomized, controlled, 20-week-long high-intensity strength and sprint training trial in middle-aged and old male sprint athletes—an aged group likely to participate in this kind of vigorous exercise–Suominen et al. (2017) reported significant albeit modest improvements in the mid-tibial (not distal) structure and strength, which were most pronounced in the more compliant athletes. This indicates that novel, intensive load-bearing, even of short duration can strengthen aging bones, even in subjects with a long-term high-impact training background [107••]. These data confirm a degree of mechanoadaptability in aging bones and imply that very high load stimuli may prime responses even in the aged [107••]. Further support for this notion comes from an examination of a human calcaneal trabecular bone, which experiences extreme repetitive forces during endurance running. Accordingly, Best et al. (2017) recruited forefoot- and rearfoot-striking runners, and non-runners and, having confirmed strike pattern using a motion capture system, reported greatest mean trabecular thickness and BMD in forefoot runners which correlated positively with weekly running distance and “running years” and negatively with age at running onset. As trabecular thickness, BMD and BV/TV were highly correlated with body mass in non-runners; adjustment for body mass revealed that individuals exposed to the greatest summative load stimulus (from running) had the thickest trabeculae, leading the authors to conclude that early adoption and years of sustained running promotes trabecular bone accrual in the posterior calcaneus [108]. This is supported by a more recent study reporting that moderate-to-vigorous physical activity protects against age-related decline in bone mass in a “dose” and daily-pattern dependent manner [109]. Perhaps mechanoadaptive responses can indeed exert effects on bone mass and architecture, when primed appropriately, even in the aged.

This proposition was addressed by Sundh et al. (2018) who recruited 20 healthy and inactive postmenopausal women into a 3-month-long exercise program of daily, one-legged, high-impact jumps (“jump-loading”, contralateral control) after which bone microarchitecture and mid-tibial bone material strength index were measured with a handheld indentation instrument. They reported that the material strength index increased by 7% with jump-loading, relative to the control, without affecting bone microstructure, geometry, or density leading to a conclusion that unilateral high-impact mechanical loading was indeed mechanoadaptive in the aged and could rapidly improve bone material properties even before any overt changes in bone mass or structure [110].

Clues to the precise quality of this high-impact stimulus that is beneficial for aged bone may come from studies linking bone biomechanical properties and physical activity-related load history. Niinimäki et al. (2017), for instance, studied bone properties including bending and torsional strength, cortical area, direction of the major axis (theta angle), and shape ratios in cross-sections of the hip and proximal femur, collected by MRI from female Finnish athletes engaged in a range of sporting activities (high-jump, triple-jump, endurance running, swimming, power-lifting, soccer and squash and, a group of active non-athletes). They found that triple-jumpers and soccer and squash players had the greatest cortical torsional strength, swimmers and non-athletes the smallest, whereas high-jumpers, power-lifters, and endurance runners exhibited interim values. This lead the authors to conclude that extreme, directionally inconsistent loading necessitate a more robust skeleton compared with directionally consistent or non-impact loads [111]. The work by Giarmatzis et al. (2017) indicated that the elderly exhibit smaller stride length and hip adduction angle at peak loading during both walking and running [112] and suggests that an exercise regime initially aimed at reversing these trends may be beneficial in preventing age-related hip bone loss.

## Priming to Restore Adaptive Responses

The origins of such priming events that restore bones’ mechanoadaptive response to loading in the aged skeleton remain ill-defined. Whether they might be instigated by specific short-term modifications in the mechanical loading environment remains almost entirely unexplored. Clues to the identity of these mechanical priming events in the aged bone may emerge from the elucidation of the mechanisms underpinning the response to specific loads engendered by high impact exercise. To address whether mechanoadaptation fails in aged mice due to a strain thresholding effect and whether exceeding this threshold can act as a priming stimulus, a recent study by Javaheri et al. (2018) explored whether imposition of a brief high-magnitude, load-priming regime might restore mechanoadaptive responses in both cortical and trabecular bone of aged female mice. In addition, the authors sought to identify the mechanisms involved by spatial correlation with known regulators of bone accrual. The authors reported that two bouts of additional high magnitude load, producing local strains more than double of those capable of eliciting a mechanoadaptive response in young mice (~ 5500 με), effectively primed a mechanoadaptive response in the cortical but not the trabecular bone in aged mice to subsequent lower magnitude loading bouts. This “priming” function was implicated on the basis that these latter, low-magnitude loading bouts engendered strains which only matched those which the bone was otherwise unresponsive in other groups of aged mice. Intriguingly, this load-priming of increased cortical mechanoadaptive bone accrual in aged mice was regionally-correlated with the down-regulation of osteocyte Sclerostin expression, which serves as a proxy measure of osteocyte response to load. These data suggest that the failure of aged bone to adapt in response to loading is due to the evoking of an insufficient mechanical stimulus and/or a dysfunctional osteocyte-mediated mechanotransduction of this stimulus, either of which can be reactivated in aged bones by short bursts of high magnitude loading [86••].

## New Concept

It, therefore, remains unclear whether aged bones lose their responsiveness to mechanical loading due to a failure of the osteocytes to sense load, a failure of osteoblasts to lay down new bone, or insufficient mechanical stimulus to trigger a response. We and others have shown that mechanoadaptive responses to load are deficient in the aged bone (despite induction of relatively high strains) but can be restored by the imposition of either disuse or supra-physiological high-magnitude loads. These findings imply that loading, which is clearly mechanoadaptive in young bones, generates a divergent mechanical stimulus in the aged bone, which fails to elicit a response. We hypothesize that aged bone mechanoadaptation is not driven solely by the magnitude of strain but is instead sensitive to local high strain gradients. Deciphering the factor(s) underpinning these shifts in mechanoadaptive capacity will enable new approaches for retaining bone health in the aged. Exercise is a commonly recommended intervention for preventing bone fragility, and perhaps, its enlightenment in light of these in vivo studies is now a profound prospect.

**Fig. 1:**
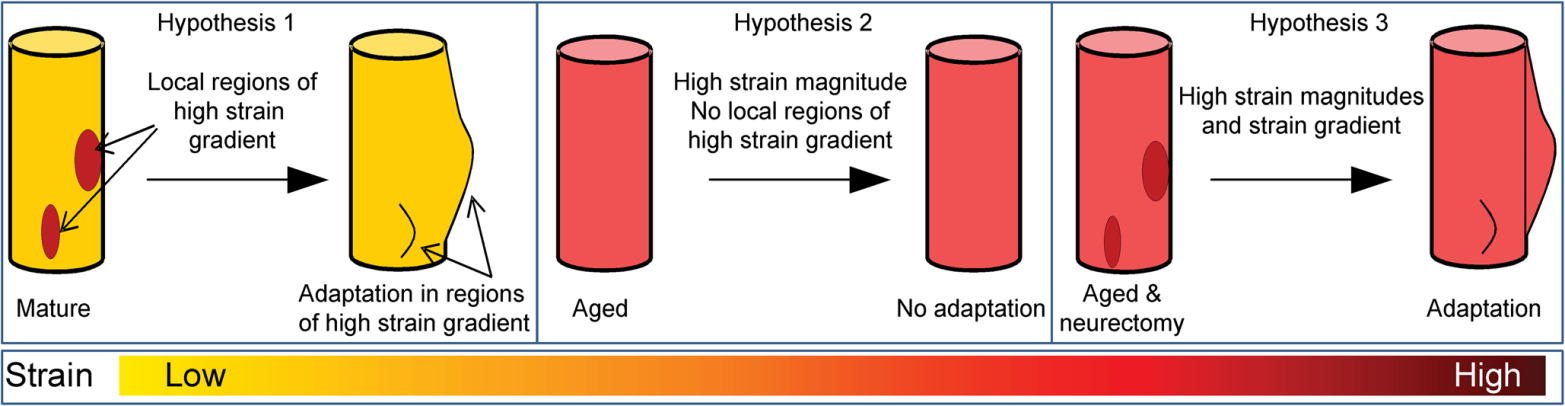
We hypothesize that (1) bone formation in the cortex occurs in regions of high strain gradients; (2) regions of high strain gradient are absent in aged cortical bones; and that (3) aged cortical bone can be “kick-started” for osteogenic activity by inducing regions of high strain gradient. It is presumed that trabecular bone may utilize similar mechanisms

Recent studies provide more guidance on the effectiveness of exercise regimes to restore the age-related decline in bone adaptive responses, emphasizing the notion that only high-impact exercise regimes are osteogenic. Innovative exercise paradigms appear to be capable of “hacking” into the osteogenic signal produced by exercise such that low-to-moderate intensity activities may also become more beneficial. It is tempting therefore for us to speculate that bone formation occurs in regions of high strain gradients, that regions of high strain gradient are absent in aged bones, and that aged bone can be “kick-started” for osteogenic activity by the transient induction of regions of high strain gradient (Fig. 1).

## Conclusions

The growing skeleton experiences a diversity of mechanical loads that modulate the accrual of bone mass and hence bone strength. However, with increasing age, there is a failure to maintain the balance between formation and resorption with resultant, progressive net bone loss. Previous studies by us and others have documented that the bone’s adaptive response to anti-resorptive/anabolic stimulation by mechanical loading is impaired and that innovative loading strategies have the potential to prevent some of the deleterious effects of aging on bones and restore the functionally-relevant structure in the elderly to prevent age-related bone loss and osteoporosis.
